# Sex differences in the relationships between 24-h rest-activity patterns and plasma markers of Alzheimer’s disease pathology

**DOI:** 10.1186/s13195-024-01653-y

**Published:** 2024-12-30

**Authors:** Maxime Van Egroo, Elise Beckers, Nicholas J. Ashton, Kaj Blennow, Henrik Zetterberg, Heidi I. L. Jacobs

**Affiliations:** 1 Faculty of Health, Medicine and Life Sciences, Mental Health and Neuroscience Research Institute, Alzheimer Centre Limburg, Maastricht University, Maastricht, The Netherlands; 2 Athinoula A. Martinos Center for Biomedical Imaging, Department of Radiology, Massachusetts General Hospital, Harvard Medical School, Boston, MA USA; 3 Sleep and Chronobiology Lab, CRC-In Vivo Imaging Unit, GIGA-Institute, University of Liège, Liège, Belgium; 4 Department of Psychiatry and Neurochemistry, Institute of Neuroscience and Physiology, The Sahlgrenska Academy at the University of Gothenburg, Mölndal, Sweden; 5 Centre for Age-Related Medicine, Stavanger University Hospital, Stavanger, Norway; 6 Institute of Psychiatry, Psychology and Neuroscience, Maurice Wohl Institute Clinical Neuroscience Institute, King’s College London, London, Maurice UK; 7 NIHR Biomedical Research Centre for Mental Health and Biomedical Research Unit for Dementia at South London and Maudsley NHS Foundation, London, UK; 8 Clinical Neurochemistry Laboratory, Sahlgrenska University Hospital, Mölndal, Sweden; 9 Pitié-Salpêtrière Hospital, Paris Brain Institute, ICM, Sorbonne University, Paris, France; 10 Neurodegenerative Disorder Research Center, Division of Life Sciences and Medicine, Department of Neurology, Institute on Aging and Brain Disorders, University of Science and Technology of China and First Affiliated Hospital of USTC, Hefei, P.R. China; 11 Department of Neurodegenerative Disease, UCL Institute of Neurology, Queen Square, London, UK; 12 UK Dementia Research Institute at UCL, London, UK; 13 Hong Kong Center for Neurodegenerative Diseases, Hong Kong, China; 14 Wisconsin Alzheimer’s Disease Research Center, School of Medicine and Public Health, University of Wisconsin, University of Wisconsin-Madison, Madison, WI USA; 15 UNS40 box 34, P.O. Box 616, Maastricht, 6200 MD The Netherlands

**Keywords:** 24-h rest-activity patterns, Actigraphy, Amyloid-beta, Glial fibrillary acidic protein, Interdaily stability, Intradaily variability, Neurofilament light chain, Plasma biomarkers, Sex differences, Tau

## Abstract

**Background:**

Although separate lines of research indicated a moderating role of sex in both sleep-wake disruption and in the interindividual vulnerability to Alzheimer’s disease (AD)-related processes, the quantification of sex differences in the interplay between sleep-wake dysregulation and AD pathology remains critically overlooked. Here, we examined sex-specific associations between circadian rest-activity patterns and AD-related pathophysiological processes across the adult lifespan.

**Methods:**

Ninety-two cognitively unimpaired adults (mean age = 59.85 ± 13.77 years, range = 30–85, 47 females) underwent 10 days of actigraphic recordings, and blood drawing. Standard non-parametric indices of 24-h rest-activity rhythm fragmentation (intradaily variability, IV) and stability (interdaily stability, IS) were extracted from actigraphy data using the *GGIR* package. Plasma concentrations of neurofilament light chain (NfL), glial fibrillary acidic protein (GFAP), amyloid-β_42/40_ (Aβ_42/40_), total tau, and tau phosphorylated at threonine 181 (p-tau_181_) or threonine 231 (p-tau_231_) were measured using Single molecule array technology. Multiple linear regression models were adjusted for age, sex, education, body mass index, and actigraphic recording duration.

**Results:**

Higher IV, indicating worse 24-h rest-activity rhythm fragmentation, was associated with elevated levels of plasma NfL (*t*(85) = 4.26, *P* < 0.0001), GFAP (*t*(85) = 2.49, *P* = 0.01), and at trend level with lower Aβ_42/40_ ratio values (*t*(85) = -1.95, *P* = 0.054). Lower IS, reflecting less day-to-day stability in the 24-h rest-activity rhythm, was linked to elevated levels of plasma NfL (*t*(85) = -2.24, *P* = 0.03), but not with the other plasma biomarkers. Importantly, interaction models demonstrated that male participants were driving the observed relationships between IV and plasma NfL (*t*(84) = 4.05, *P* < 0.001) or GFAP (*t*(84) = 3.60, *P* < 0.001), but also revealed a male vulnerability in models testing interactions with p-tau_181_ (IV: *t*(76) = 3.71, *P* < 0.001; IS: *t*(76) = -3.30, *P* = 0.001) and p-tau_231_ (IV: *t*(82) = 3.28, *P* = 0.002). Sensitivity analyses further showed that accounting for potential confounding factors such as *APOE* genotype, depression, and self-reported symptoms of possible sleep apnea did not modify the observed relationships.

**Conclusions:**

These findings suggest that the association between disrupted circadian rest-activity patterns and AD pathophysiological processes may be more evident in cognitively unimpaired males. Our results contribute to the precision medicine approach, and they have clinical implications for improved early detection and selection of at-risk individuals to be enrolled in preventive interventions.

**Supplementary Information:**

The online version contains supplementary material available at 10.1186/s13195-024-01653-y.

## Background

Over the past decade, circadian rhythm disturbances and sleep-wake dysregulation have been established as important risk factors contributing to the unfolding of hallmark Alzheimer’s disease (AD) pathophysiological processes, as early as in the preclinical stages of the disease [1, 2]. In asymptomatic older individuals, alteration in the 24-h rest-activity rhythm, a proxy measure of the circadian organization of the sleep-wake cycle, and poor sleep quality have been linked to higher amyloid-beta (Aβ) and tau burden [3–6], likely in a bidirectional manner [7], as well as to widespread brain grey and white matter changes [8–11]. Furthermore, a large longitudinal cohort study demonstrated that increasing fragmentation and instability of the 24-h rest-activity rhythm was associated with worsening of cognitive performances and clinical progression across the AD continuum in older adults devoid of dementia at baseline [12].

Importantly, previous research has highlighted sex differences in the nature and magnitude of sleep and circadian rest-activity rhythm disturbances in older adults, but also when examining the interindividual vulnerability to AD pathology and risk of developing AD dementia. On the one hand, one common observation is that men exhibit more sleep-wake disruption compared to women when sleep-wake quality is investigated with objective measurements (e.g., polysomnography (PSG) and actigraphy), whereas women report greater sleep-wake disturbances than men when measured through subjective questionnaires [12–17]. On the other hand, evidence from autopsy and in vivo studies suggests that, compared to their male counterparts, older women show higher tau burden [18–20], particularly in the context of elevated Aβ levels [21], as well as worse tau-dependent cognitive impairment and faster rates of tau-dependent cognitive decline [22, 23], and increased risk of developing AD dementia at younger ages in *APOE* ε4 carriers [24].

Surprisingly, although sex constitutes a common factor differentially involved in both sleep-wake disruption and AD-related processes, hardly any research examined the moderating effect of sex on the connection between these variables. Instead, most studies included sex as a covariate in their statistical models linking sleep-wake indices to AD measures, but this practice does not inform about potential sex differences in the highlighted associations. In the ongoing effort to adopt a precision medicine approach, elucidating the role of sex in the interplay between sleep-wake dysregulation and AD pathogenesis constitutes a crucial step for the improved identification of individuals at risk for AD trajectories and for the future development of personalized preventive strategies early on in the disease course [2, 25].

Here, we used actigraphy to objectively characterize 24-h rest-activity rhythms and we assessed plasma AD-related pathological markers in a sample of cognitively unimpaired adults across the lifespan. The objectives of the present study were twofold: first, we sought to highlight novel associations between circadian rest-activity patterns and AD pathophysiological processes by leveraging recent advances in blood-based AD biomarkers. Second, we aimed to explore potential sex differences that may drive –or mask– the observed relationships.

## Methods

### Participants

Ninety-two cognitively unimpaired individuals (mean age = 59.85 ± 13.77 years, age range = 30–85 years, 47 females (51.09%) were recruited from the general community via advertisements in the Southern region of the Netherlands. The main exclusion criteria were: performance on Rey-Auditory Verbal Learning Test two standard deviations below the mean (according to normative data corrected for age, sex, and education), Mini-Mental State Examination scores < 26, shift working, history of major psychiatric or neurological disorders, history of brain injury or brain surgery, left-handedness, use of medications that may influence cognitive functioning, excessive alcohol consumption (> 15 units/week), and possible depression or depressive symptoms (Hamilton Depression Rating Scale, all individuals within normal range = 0–12, mean = 2.18 ± 2.47).

### Plasma biomarkers and *APOE* genotyping

Fasted EDTA plasma samples were obtained through venipuncture from the antecubital vein. Within 60 min of collection, samples were centrifuged at 2000×*g*, aliquoted in polypropylene tubes, and stored at − 80 °C in the central biobank of Maastricht University Medical Center. Plasma samples were analyzed in randomized order using ultra-sensitive Single molecule array (Simoa) assays to measure plasma levels of total tau (t-tau, Neurology 3-Plex A Advantage Kit, Quanterix, Inc), tau phosphorylated at threonine 181 (p-tau_181_, pTau-181 V2 Advantage Kit, Quanterix, Inc), tau phosphorylated at threonine 231 (p-tau_231_, University of Gothenburg), neurofilament light chain (NfL, University of Gothenburg), glial fibrillary acidic protein (GFAP, University of Gothenburg), as well as Aβ_42_ and Aβ_40_ (University of Gothenburg) which were used to compute the Aβ_42/40_ ratio. Analyses were performed in duplicates using a 1:4 automated dilution protocol for all markers, except for 1:2 dilution protocol for p-tau_231_. *APOE* genotyping was performed using polymerase chain reaction on DNA extracted from whole blood samples. Participants were considered ‘ε4 carriers’ if they carry at least one ε4 allele. In the whole sample, 33 individuals were *APOE* ε4 carriers, with 28 carriers of one ε4 allele and 5 carriers of two ε4 alleles. Technicians handling the blood samples were blinded to the participant demographic and actigraphy data, and staff members collecting actigraphy data were blinded to plasma biomarkers and *APOE* genotyping results.

### Actigraphic recordings

Within on average 1.29 ± 0.31 years following the blood draw, participants underwent ten days of continuous actigraphic recording using a tri-axial accelerometer (Axivity AX3 device, Axivity Ltd, Newcastle, UK) worn on their non-dominant wrist (sampling rate = 100 Hz, sampling range = ± 8 g). Actigraphic recordings were processed using the open-source *GGIR* package [26] (version 2.8.2, https://CRAN.R-project.org/package=GGIR) implemented in R (version 4.1.1). For all participants, quality of the actigraphy data was checked by careful visual inspection of raw actigraphic recordings in the OMGUI software (version 1.0.0.43, https://github.com/openmovementproject/openmovement), as well as through the different quality indicators (e.g., calibration results, detection of corrupted recordings) and visualization plots of acceleration time series automatically generated by the *GGIR* package. Standard non-parametric indices of 24-h rest-activity rhythm fragmentation (intradaily variability, IV) and stability (interdaily stability, IS) were computed in *GGIR* following the original approach proposed by van Someren et al. [27]. IV values represent the hourly variability in the occurrence of rest vs. activity periods and are higher in individuals with more frequent daytime sleep periods or nocturnal awakenings, whereas IS values reflect synchronization of an individual’s 24-h rest-activity rhythm with environmental zeitgebers (“time givers”), such as the light-dark cycle, and are higher in individuals with a more stable day-to-day profile of rest-activity rhythm [27, 28]. In addition to the actigraphic recording, participants also filled in an extended version of the Groningen Sleep Quality Scale [29], which included four dichotomous items related to common symptoms of sleep disordered breathing, i.e., snoring, waking up in the morning with a sore throat, waking up in the morning with a dry mouth, and waking up in the morning with a headache. Based on these four items, a sum score of self-reported possible sleep apnea was computed and used in sensitivity analyses.

### Statistical analyses

Statistical analyses were performed in R (version 4.1.1, www.r-project.org). First, multiple linear regression models adjusted for demographic variables (age, sex, education, and body mass index (BMI)) and actigraphic recording duration were used to investigate the relationships between plasma AD biomarkers levels and actigraphy-derived 24-h rest-activity rhythm fragmentation (IV) or stability (IS). In a second step, interaction models were performed to examine sex differences in these associations. Sensitivity analyses were conducted by including additional potential confounding factors to the previous models, including *APOE* genotype, depression, and self-reported symptoms of possible sleep apnea. For the interaction models, an additional sensitivity analysis was conducted by further stratifying the group of women into younger vs. older women using a median split based on age. Two individuals had outlier values (> 4 standard deviations above the mean of the sample) on plasma total tau, p-tau_181_ and p-tau_231_ levels only and were therefore excluded from analyses involving these tau-related variables. The threshold for statistical significance was set at two-sided *P* < 0.05. Correction for multiple comparisons in the main analyses was performed using the False Discovery Rate (FDR) approach per actigraphy metric.

## Results

### Associations between demographics and actigraphy-derived 24-h rest-activity rhythm metrics or plasma AD biomarkers

Demographic characteristics, actigraphic variables, and plasma AD biomarkers levels in the study sample are detailed in Table 1. In this cohort of cognitively unimpaired individuals across the adult lifespan, older age was significantly associated with higher IS (*t*(86) = 3.53, *P* < 0.001) and showed a marginal association with higher IV (*t*(86) = 1.87, *P* = 0.06). Male participants tended to display lower IS (*t*(86) = -1.82, *P* = 0.07) and had significantly higher IV (*t*(86) = 2.57, *P* = 0.01, Supplementary Table 1). With regards to plasma AD biomarkers, older age was associated with elevated levels of NfL (*t*(87) = 8.03, *P* < 0.0001), GFAP (*t*(87) = 5.31, *P* < 0.0001), p-tau_181_ (*t*(79) = 2.58, *P* = 0.01), and with lower values of Aβ_42/40_ ratio (*t*(87) = -3.13, *P* = 0.002). Male participants had higher levels of plasma p-tau_181_ (*t*(79) = 2.20, *P* = 0.03), but did not significantly differ from female participants on the other plasma measures. As recently reported in the BioFINDER study [30], higher BMI was related to lower levels of plasma NfL (*t*(87) = -2.04, *P* = 0.04) and GFAP (*t*(87) = -2.20, *P* = 0.03, Supplementary Table 2).

**Table 1:** Demographic characteristics, actigraphic variables, and plasma Alzheimer’s disease-related biomarkers in the whole study sample (*n* = 92), as well as stratified by sex

	Whole sample (*n* = 92)	Females (*n* = 47)	Males (*n* = 45)	*P* value*
**Demographic characteristics**				
Age, years	59.85 ± 13.77 [30–85]	58.15 ± 13.48 [30–80]	61.62 ± 14.01 [31–85]	0.24
Education, years	14.52 ± 1.99 [10–20]	14.34 ± 2.08 [10–20]	14.71 ± 1.89 [10–20]	0.24
Ethnicity	Caucasian	Caucasian	Caucasian	
Right-handed, *n* (%)	92 (100)	47 (100)	45 (100)	
Body mass index, Kg/m²	24.45 ± 2.95 [17.56–31.71]	23.92 ± 3.12 [17.56–31.71]	25.01 ± 2.68 [19.71–30.04]	0.08
*APOE* ε4 carriers, *n* (%)	33 (36)	14 (30)	19 (42)	0.28
One allele, *n* (%)	28 (30)	12 (26)	16 (35)	0.37
Two alleles, *n* (%)	5 (6)	2 (4)	3 (7)	0.67
Mini-Mental State Examination, score	29.00 ± 1.15 [26–30]	29.19 ± 1.01 [26–30]	28.80 ± 1.25 [26–30]	0.14
Hamilton Depression Rating Scale, score	2.18 ± 2.47 [0–12]	2.36 ± 2.75 [0–12]	2.00 ± 2.14 [0–11]	0.82
Groningen Sleep Quality Scale, score	2.87 ± 3.46 [0–14]	3.68 ± 3.60 [0–13]	2.10 ± 3.17 [0–14]	**0.02**
Self-reported sleep apnea symptoms, score	0.70 ± 0.86 [0–3]	0.80 ± 0.97 [0–3]	0.60 ± 0.73 [0–3]	0.47
Self-reported use of sleep medication, *n* (%)^a^	7 (8)	5 (11)	2 (4)	0.10
**Actigraphic variables**				
Recording duration, days	9.80 ± 0.73 [7.07–12.07]	9.71 ± 0.59 [7.80–11.55]	9.89 ± 0.85 [7.07–12.07]	0.52
Actigraphic device wear ratio	1.00 ± 0.004 [0.97–1.00]	1.00 ± 0.005 [0.97–1.00]	1.00 ± 0.003 [0.99–1.00]	0.54
Intradaily variability	0.53 ± 0.16 [0.27–1.08]	0.48 ± 0.12 [0.27–0.77]	0.58 ± 0.18 [0.33–1.08]	**0.005**
Interdaily stability	0.63 ± 0.09 [0.43–0.83]	0.65 ± 0.09 [0.44–0.83]	0.61 ± 0.08 [0.43–0.77]	0.07
**Plasma AD biomarkers**				
NfL (pg/ml)	18.6 ± 7.32 [6.56–43.4]	18 ± 6.77 [6.78–34.6]	19.2 ± 7.88 [6.56–43.4]	0.49
GFAP (pg/ml)	153 ± 60.2 [53.8–389]	156 ± 60.3 [53.8–336]	149 ± 60.6 [75.3–389]	0.37
Aβ_40_ (pg/ml)	92.1 ± 11.3 [66.3–125]	91.4 ± 9.18 [73.7–119]	92.9 ± 13.2 [66.3–125]	0.53
Aβ_42_ (pg/ml)	8.35 ± 1.30 [3.32–11]	8.36 ± 1.24 [5.98–11.02]	8.34 ± 1.37 [3.32–10.8]	0.94
Aβ_42/40_ ratio	0.09 ± 0.01 [0.05–0.12]	0.09 ± 0.01 [0.06–0.12]	0.09 ± 0.01 [0.05–0.12]	0.62
t-tau (pg/ml)^b^	2.63 ± 0.87 [0.75–7.58]	2.68 ± 0.71 [0.75–4.01]	2.58 ± 1.02 [1.39–7.58]	0.18
p-tau_181_ (pg/ml)^b^	1.66 ± 0.75 [0.80–6.44]	1.48 ± 0.55 [0.80–3.15]	1.85 ± 0.89 [0.91–6.44]	**0.004**
p-tau_231_ (pg/ml)	8.39 ± 3.80 [3.29–28.9]	7.75 ± 2.59 [3.81–16.3]	9.07 ± 4.68 [3.29–28.9]	0.33

^a^ data missing for *n* = 10, ^b^ data missing for *n* = 6

*P values related to statistical differences between female and male participants were computed using χ² or Fisher’s exact tests for categorical variables and Student’s *t* or Mann-Whitney *U* test for continuous variables, depending on the normality of the distribution of the variables. Abbreviations: Aβ_42/40_ = amyloid-beta_42/40_ ratio, *APOE* = Apolipoprotein E, GFAP = glial fibrillary acidic protein, NfL = neurofilament light chain, t-tau = total tau, p-tau_181_ = tau phosphorylated at threonine 181, p-tau_231_ = tau phosphorylated at threonine 231

### Associations between 24-h rest-activity rhythm fragmentation or instability and plasma AD-related biomarkers

**Fig. 1:**
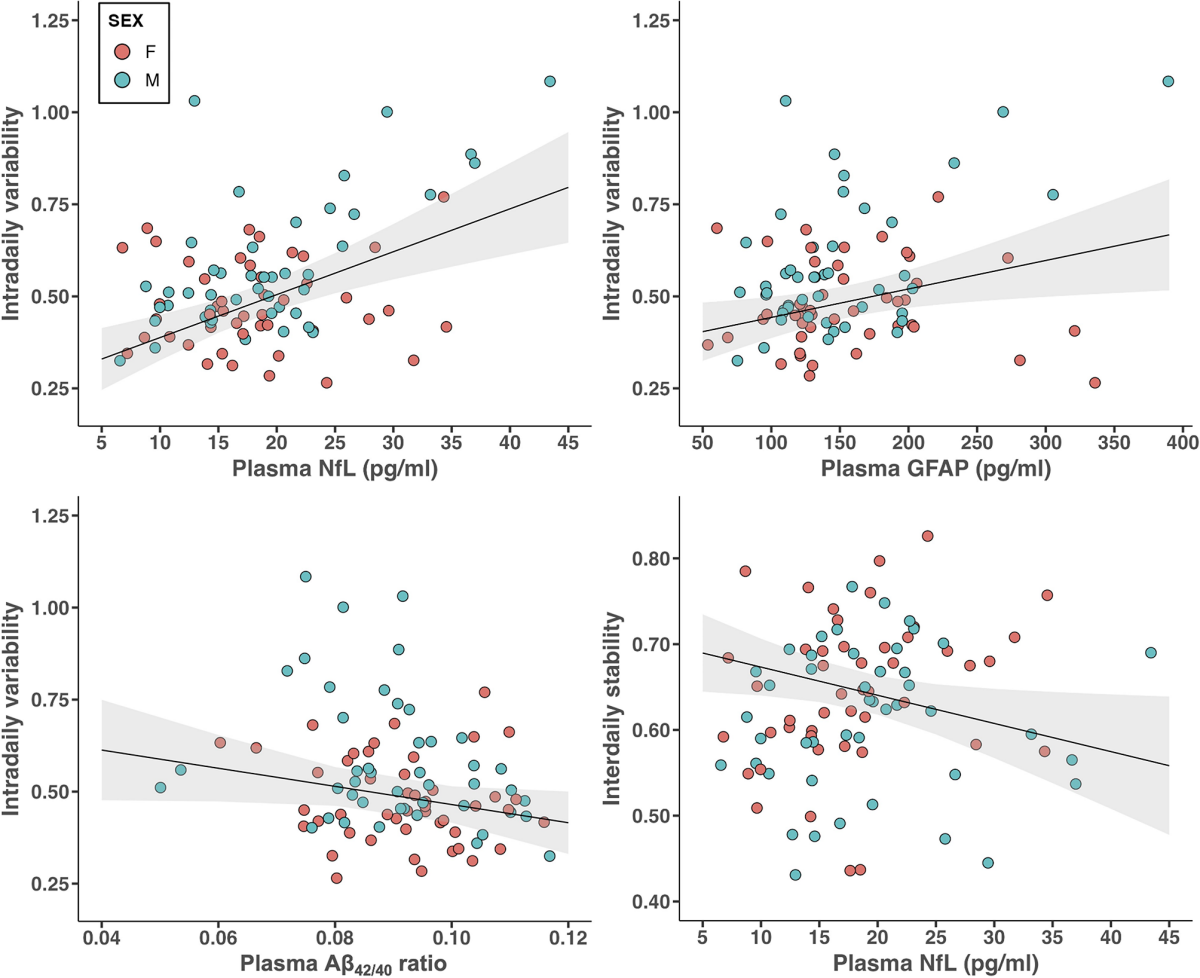
Relationships between actigraphy-derived 24-h rest-activity rhythm fragmentation (intradaily variability, IV) and plasma levels of neurofilament light chain (NfL), glial fibrillary acidic protein (GFAP), and amyloid-beta_42/40_ ratio (Aβ_42/40_). Statistical models include covariates of age, sex, education, body mass index, and actigraphic recording duration. Red circles = female participants, blue circles = male participants

After adjusting for demographic variables and actigraphic recording duration, multiple linear regression models showed that higher IV, reflecting worse fragmentation of the 24-h rest-activity rhythm, was significantly associated with elevated levels of plasma NfL (*t*(85) = 4.26, *P* < 0.0001), GFAP (*t*(85) = 2.49, *P* = 0.01), and at trend level with lower Aβ_42/40_ ratio values (*t*(85) = -1.95, *P* = 0.054). By contrast, no significant associations were found with plasma levels of total tau (*t*(77) = 0.98, *P* = 0.33), p-tau_181_ (*t*(77) = -0.54, *P* = 0.59), or p-tau_231_ (*t*(83) = 0.46, *P* = 0.65). Similarly, lower IS, indicating greater 24-h rest-activity rhythm instability, was significantly associated with elevated levels of plasma NfL (*t*(85) = -2.24, *P* = 0.03), but not with the other plasma biomarkers (Table 2; Fig. 1). Sensitivity analyses further showed that including additional potential confounding factors of *APOE* genotype, depression, and self-reported symptoms of possible sleep apnea did not change these findings (Supplementary Tables 3–5). After correction for multiple comparisons, only the associations between IV and plasma levels of NfL (*P*_FDR_ = 0.0003) or GFAP (*P*_FDR_ = 0.04) remained significant.

**Table 2:** Statistical outputs of the multiple linear regression models investigating the main effects of plasma Alzheimer’s disease-related biomarkers (predictors) on outcome measures of actigraphy-derived 24-h rest-activity rhythm fragmentation (intradaily variability, top) or stability (interdaily stability, bottom) in the whole study sample (*n* = 92). Abbreviations: Aβ_42/40_ = amyloid-beta_42/40_ ratio, AD = Alzheimer’s disease, GFAP = glial fibrillary acidic protein, NfL = neurofilament light chain, t-tau = total tau, p-tau_181_ = tau phosphorylated at threonine 181, p-tau_231_ = tau phosphorylated at threonine 231

	Intradaily variability												
	NfL		GFAP		Aβ_42/40_		t-tau		*p*-tau_181_		*p*-tau_231_		
	*t*	*P*	*t*	*P*	*t*	*P*	*t*	*P*	*t*	*P*	*t*	*P*	
Plasma AD biomarker	4.26	**< 0.0001**	2.49	**0.01**	-1.95	0.054	0.98	0.33	-0.54	0.59	0.46	0.65	
Age	-1.21	0.23	0.43	0.67	1.18	0.24	1.24	0.22	1.23	0.22	1.44	0.15	
Male sex	2.69	**0.009**	2.84	**0.006**	2.59	**0.01**	2.30	**0.02**	2.24	**0.03**	2.29	**0.02**	
Education	0.85	0.40	1.01	0.32	0.80	0.43	0.89	0.38	0.80	0.42	0.97	0.34	
Body mass index	0.86	0.39	0.59	0.57	0.06	0.96	0.61	0.55	0.54	0.59	0.77	0.45	
Actigraphic recording duration	1.61	0.11	1.86	0.07	1.67	0.10	2.65	**0.01**	2.83	**0.006**	1.94	0.06	
	**Interdaily stability**												
	**NfL**		**GFAP**		**Aβ**_**42/40**_		**t-tau**		**p-tau**_**181**_		**p-tau**_**231**_		
	***t***	***P***	***t***	***P***	***t***	***P***	***t***	***P***	***t***	***P***	***t***	***P***	
Plasma AD biomarker	-2.24	**0.03**	-0.60	0.55	-0.12	0.91	-0.23	0.82	-0.54	0.59	-0.73	0.47	
Age	4.20	**< 0.001**	5.36	**< 0.001**	3.30	**0.001**	3.18	**0.002**	3.26	**0.002**	3.29	**0.001**	
Male sex	-1.80	0.08	-1.86	0.07	-1.81	0.07	-1.74	0.09	-1.57	0.12	-1.82	0.07	
Education	-1.95	0.06	-1.94	0.06	-1.90	0.06	-2.03	**0.05**	-2.01	**0.05**	-2.11	**0.04**	
Body mass index	-1.34	0.18	-1.00	0.32	-0.88	0.38	-1.15	0.25	-1.23	0.22	-1.12	0.27	
Actigraphic recording duration	-1.74	0.09	-1.84	0.07	-0.12	0.91	-1.82	0.07	-1.76	0.08	-1.66	0.10	

### Sex differences in the relationships between 24-h rest-activity rhythm fragmentation or instability and plasma AD-related biomarkers

**Fig. 2:**
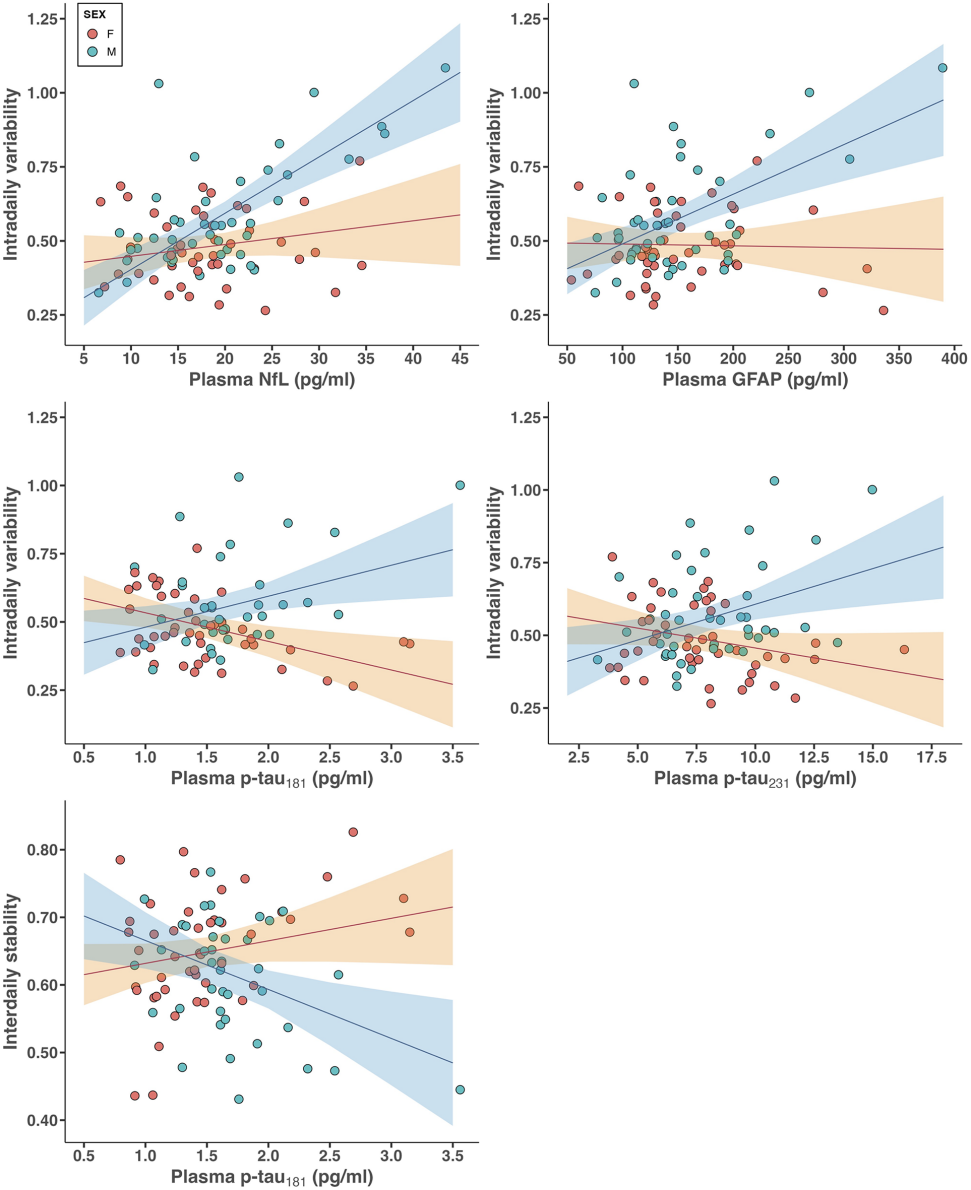
Sex differences in the associations between actigraphy-derived 24-h rest-activity rhythm fragmentation (intradaily variability, IV) or stability (interdaily stability, IS) and plasma levels of neurofilament light chain (NfL), glial fibrillary acidic protein (GFAP), tau phosphorylated at threonine 181 (p-tau_181_), and tau phosphorylated at threonine 231 (p-tau_231_). Statistical models include covariates of age, sex, education, body mass index, and actigraphic recording duration. Regression lines and associated 95% confidence interval for female and male participants are displayed in red and blue, respectively

In a second step, we performed interaction models to investigate potential sex differences in the observed relationships. Our results demonstrate that the positive associations between IV values and plasma NfL or GFAP levels were mainly driven by male participants (interaction NfL*Sex: *t*(84) = 4.05, *P* < 0.001; interaction GFAP*Sex: *t*(84) = 3.60, *P* < 0.001, Table 3; Fig. 2). Of note, this analysis further revealed that higher IV was associated with elevated levels of p-tau_181_ (*t*(76) = 3.71, *P* < 0.001) and p-tau_231_ (*t*(82) = 3.28, *P* = 0.002) in males as compared to females. By contrast, no significant interactions were found when examining the associations with plasma Aβ_42/40_ ratio (*t*(84) = -1.01, *P* = 0.32) or total tau (*t*(76) = 0.36, *P* = 0.72). Similar interaction patterns were present in models considering IS, with only males showing a negative association between IS and plasma p-tau_181_ (*t*(76) = -3.30, *P* = 0.001). In addition, all the significant interactions remained unchanged after further adjusting for interactions between sex and *APOE* genotype, depression, or self-reported symptoms of possible sleep apnea (Supplementary Tables 6–8), and they survived correction for multiple comparisons. Finally, interaction models further stratifying the group of women into younger vs. older women yielded similar results (Supplementary Table 9, Supplementary Fig. 1).

**Table 3:** Statistical outputs of the multiple linear regression models testing for sex differences (interaction effects) in the relationships between plasma Alzheimer’s disease-related biomarkers (predictors) and outcomes measures of actigraphy-derived 24-h rest-activity fragmentation (intradaily variability, top) or stability (interdaily stability, bottom). Abbreviations: Aβ_42/40_ = amyloid-beta_42/40_ ratio, AD = Alzheimer’s disease, GFAP = glial fibrillary acidic protein, NfL = neurofilament light chain, t-tau = total tau, p-tau_181_ = tau phosphorylated at threonine 181, p-tau_231_ = tau phosphorylated at threonine 231

	Intradaily variability												
	NfL		GFAP		Aβ_42/40_		t-tau		*p*-tau_181_		*p*-tau_231_		
	*t*	*P*	*t*	*P*	*t*	*P*	*t*	*P*	*t*	*P*	*t*	*P*	
Plasma AD biomarker*Male sex	4.05	**< 0.001**	3.60	**< 0.001**	-1.01	0.32	0.36	0.72	3.71	**< 0.001**	3.28	**0.002**	
Plasma AD biomarker	1.26	0.21	-0.16	0.87	-0.60	0.55	0.54	0.59	-2.78	**0.007**	-1.76	0.08	
Age	-1.75	0.09	0.30	0.77	1.11	0.27	1.26	0.21	1.82	0.07	1.78	0.08	
Male sex	-2.65	**0.01**	-2.17	**0.03**	1.37	0.17	0.28	0.78	-2.74	**0.008**	-2.37	**0.02**	
Education	0.87	0.39	0.86	0.39	0.88	0.38	0.92	0.36	0.95	0.35	1.03	0.31	
Body mass index	1.02	0.31	0.77	0.44	0.27	0.79	0.58	0.56	0.39	0.70	0.87	0.39	
Actigraphic recording duration	1.46	0.15	2.29	**0.02**	1.65	0.10	2.59	**0.01**	2.00	**0.05**	-1.44	0.15	
	**Interdaily stability**												
	**NfL**		**GFAP**		**Aβ**_**42/40**_		**t-tau**		**p-tau**_**181**_		**p-tau**_**231**_		
	***t***	***P***	***t***	***P***	***t***	***P***	***t***	***P***	***t***	***P***	***t***	***P***	
Plasma AD biomarker*Male sex	-1.73	0.09	-1.47	0.15	-0.01	0.99	0.94	0.35	-3.30	**0.001**	-1.67	0.09	
Plasma AD biomarker	-0.77	0.45	0.44	0.66	-0.08	0.94	-0.77	0.45	1.62	0.11	0.52	0.61	
Age	4.41	**< 0.001**	3.44	**< 0.001**	3.27	**0.002**	3.25	**0.002**	2.98	**0.004**	3.18	**0.002**	
Male sex	0.92	0.36	0.64	0.52	-0.26	0.80	-1.38	0.17	2.59	**0.01**	1.02	0.31	
Education	-1.95	0.06	-1.87	0.07	-1.88	0.06	-1.93	0.06	-2.21	**0.03**	-2.13	**0.04**	
Body mass index	-1.39	0.17	-1.07	0.29	-0.86	0.39	-1.21	0.23	-1.13	0.26	-1.16	0.25	
Actigraphic recording duration	-1.63	0.11	-1.97	**0.05**	-1.80	0.08	-1.89	0.06	-0.96	0.34	-1.36	0.18	

## Discussion

Converging evidence recently established that alterations in the composition and circadian organization of the sleep-wake cycle are linked to AD pathology, most likely in a bidirectional manner [7]. Despite the documented sex differences in sleep-wake quality and AD risk, so far, the role of sex in the relationship between both remains surprisingly understudied. By leveraging recent advances in blood-based biomarkers combined with actigraphy in a cohort of asymptomatic individuals across the adult lifespan, we first provide evidence that disrupted circadian rest-activity patterns, particularly fragmentation of the 24-h rest-activity rhythm, is associated with elevated levels of plasma markers of neurodegeneration and astrogliosis, and to a lesser extent with higher Aβ burden. Crucially, males exhibited a specific vulnerability in the relationships between circadian rest-activity patterns and plasma AD biomarkers of phosphorylated tau burden, neurodegeneration, and astrogliosis. Altogether, worse fragmentation and instability of the 24-h rest-activity rhythm was associated with elevated plasma measures of AD-related pathophysiological processes in males but not in females. These findings therefore highlight the importance of considering the moderating effect of sex in studies investigating the relationship between disrupted circadian rest-activity patterns and AD pathology, and they have potential implications for improved early detection of at-risk individuals.

NfL is an intermediate filament protein that supports axonal stability, and is released into the cerebrospinal fluid (CSF) and blood following neuroaxonal damage [31]. The observed associations between worse IV or IS and elevated plasma NfL levels align with several in vivo neuroimaging studies linking actigraphy-derived 24-h rest-activity rhythm to alteration in brain tissue integrity, with the most abundant and consistent evidence for white matter disruption [8, 10, 32–34]. In addition, we observed a positive association between 24-h rest-activity rhythm fragmentation and plasma GFAP levels in vivo in humans. Plasma GFAP has been proposed as an early marker of Aβ-related astrogliosis in cognitively unimpaired individuals [35], and its clinical utility as a predictive biomarker of AD-related processes is increasingly underlined [36, 37]. Of particular interest, the interplay between sleep disruption and astrocytes activation/neuroinflammatory processes has received growing attention in the context of AD pathogenesis [38, 39], notably due to the modulating role of astrocytes in the regulation of sleep and circadian rhythm and their involvement in promoting glymphatic clearance of brain metabolites during sleep [40, 41]. Evidence from recent autopsy and animal studies revealed that sleep fragmentation was associated with higher expression of reactive –but not general– astrocyte marker genes in the dorsolateral prefrontal cortex [42], increased GFAP immunoreactivity in the hippocampus and in the locus coeruleus [43, 44], and astrogliosis in key sleep-promoting regions [45]. These previous results support the notion that the contribution of the fragmentation of the rest period (i.e., nocturnal awakenings) to IV values may underlie the association of worse 24-h rest-activity rhythm fragmentation with elevated plasma GFAP levels in our sample. Finally, although weaker, the negative association between IV and plasma Aβ_42/40_ ratio in our findings corroborates previous work linking the same actigraphy-derived metric to cortical Aβ burden assessed with PET imaging [4].

Interestingly, Lysen et al. [46] found no associations between 24-h rest-activity rhythm fragmentation or instability and plasma NfL, Aβ_40_, Aβ_42_, or total tau in a subsample of 849 asymptomatic older individuals from the Rotterdam Study cohort. Different factors may contribute to these discrepancies, including the wider age range or higher variability in plasma NfL values in our study sample, the lack of information about the proportion of *APOE* ε4 carriers in their subsample, or differences in the actigraphic device used to record 24-h rest-activity patterns as well as assays used to measure concentrations of plasma markers. Additional studies investigating the relationship between 24-h rest-activity rhythm characteristics and blood-based biomarkers in cognitively unimpaired individuals are therefore warranted to replicate our findings.

Importantly, we found a specific male vulnerability in the relationships between IV or IS values and plasma levels of NfL, GFAP, p-tau_181_, or p-tau_231_. So far, very few studies have explicitly tested for sex differences in the link between actigraphy-derived sleep-wake measures and AD-related variables, and the limited information available for sex-specific associations is often delivered as anecdotal or supplementary findings. One study reported no sex differences in the association between circadian rest-activity patterns and CSF measures of NfL, Aβ_42_, t-tau, or p-tau_181_ in mild-moderate AD patients [47], while another found a female vulnerability in the link between degradation in fractal motor activity regulation and PET-derived Aβ burden or CSF p-tau_181_/Aβ_42_ ratio in asymptomatic older individuals [48]. With regards to cognitive measures, a few studies along the disease continuum reported that males displayed amplified or specific associations between AD-related cognitive decline or clinical status and 24-h rest-activity rhythm characteristics [12, 47, 49], as well as sleep efficiency and regularity [50], although information on AD pathology was lacking. Considering the scarcity of available evidence and the mixed findings reported in the few studies specifically testing for sex differences in the relationship between sleep-wake dysregulation and AD pathophysiological processes, additional research remains needed to better characterize the modulating effect of sex on this interplay and to identify which sleep-wake metrics (actigraphy-, sleep EEG-, and/or questionnaire-derived) or AD pathological measures (PET-, CSF-, and/or plasma-derived) may be most sensitive to these sex differences.

The outcomes of the sensitivity analyses suggest that the observed relationships are independent of the previously established effects of *APOE* ε4 carriership, depression, and sleep apnea on sleep-wake measures and AD pathology [51–55]. Beyond these common confounding factors, we cannot preclude that other unmeasured mechanisms contribute to these sex differences: for example, evidence from *postmortem* investigation of the integrity of the hypothalamic suprachiasmatic nucleus (SCN), the core circadian pacemaker, suggests that males display a disproportionate age-related loss of SCN vasoactive intestinal polypeptide neurons compared to females [56]. Given the bidirectional interplay between SCN-driven circadian disturbances and AD pathology [57], it is possible that sex-specific alterations in neuronal populations essential to the regulation of the 24-h rest-activity rhythm may underlie a male vulnerability in the relationship between circadian dysregulation and AD-related pathophysiological processes. Relatedly, other contributing factors to be addressed in future studies might pertain to sex differences in the daily rhythms of clock genes [58] or in the expression of genes involved in sleep functions [59].

We observed comparatively more robust and consistent associations with IV than with IS, both when testing the main effects of plasma AD-related biomarkers but also their interactions with sex. By definition, and based on its computation, IV may reflect a higher frequency of nocturnal activity (i.e., intrusion of wakefulness during the sleep period) and/or more frequent daytime rest (i.e., intrusion of sleep during the wakefulness period). Thus, in addition to its value as an indicator of the circadian organization of rest and activity periods, IV is often used as a proxy measure of sleep-wake fragmentation. Interestingly, fragmentation of both sleep and wakefulness have been separately linked to AD pathology and clinical trajectories [60–64], and thus the IV metric may be a sensitive behavioral marker of early AD-related pathophysiological processes in asymptomatic individuals.

Our study has limitations. First, although we aimed to control for the potential presence of sleep apnea by including self-reported symptoms in our sensitivity analyses, a systematic PSG-derived assessment of the presence and magnitude of sleep apneas or other most common sleep disorders (e.g. insomnia, restless legs syndrome) would be required to more strictly confirm that the sex-specific associations observed in our study are not merely a consequence of the well-documented sex differences in sleep disorders and their proposed link with AD pathology [55, 65–67]. Second, we observed similar findings in sensitivity analyses stratifying the group of women into younger vs. older women. However, in light of the important impact of menopause-related hormonal changes on sleep and the onset of sleep disorders [68] as well as on AD pathology [21], the subsample of women should ideally be stratified according to menopause status and use of hormone replacement therapy. Third, previous PET studies highlighted a female vulnerability to the accumulation of tau pathology particularly when considered in interaction with Aβ burden or *APOE* status [21, 69], whereas we observed that males displayed higher plasma p-tau_181_ levels in the present cohort. The overall relatively low Aβ burden, the potential imbalance in the proportion of *APOE* ε4 carriers in younger vs. older participants (51% of *APOE* ε4 carriers in the younger half of the sample vs. 19% of *APOE* ε4 carriers in the older half of the sample), and the lifespan characteristic of the cohort might therefore have biased potential sex differences in plasma p-tau_181_ levels. Finally, the cross-sectional nature of our analysis prevented us from examining whether the observed moderating role of sex also applies to the relationship between longitudinal 24-h rest-activity rhythm metrics and accumulation of AD pathological hallmarks over time. Such longitudinal studies would further help disentangle the temporal ordering of events in the bidirectional relationship between disrupted circadian rest-activity patterns and accumulation of AD pathology. Likewise, the inclusion of patients along the AD continuum would help validating the observed male vulnerability in the context of later disease stages, as suggested by previous studies [47, 50].

## Conclusions

In the present cohort of cognitively unimpaired individuals across the adult lifespan, we observed a male vulnerability in the association between 24-h rest-activity rhythm fragmentation or instability and plasma AD-related biomarkers, which remained significant after accounting for common confounding factors. These findings therefore call for a more systematic assessment of the moderating effect of sex in studies investigating sleep-wake disruption and AD pathogenesis, beyond the consideration of sex as a covariate only. Our findings contribute to the advocated precision medicine approach, and they have implications for improved detection and selection of individuals at higher risk for AD-related processes to be enrolled in clinical trials.

## Electronic supplementary material

Below is the link to the electronic supplementary material.

Supplementary Material 1

## Data Availability

Participants did not explicitly consent to their data being made public and, therefore, access to their demographics, plasma biomarkers, or actigraphy data is restricted. Requests for the anonymized data should be made to Heidi I.L. Jacobs (http://www.heidijacobs.org; h.jacobs@maastrichtuniversity.nl or hjacobs@mgh.harvard.edu) and will be reviewed by an independent data access committee, taking into account the research proposal and intended use of the data. Data domains in which data collection is ongoing can only be shared under these regulations once data collection and quality assessment are completed. Requestors are required to sign a data sharing agreement to ensure participants’ confidentiality is maintained prior to the release of any data, and that procedures conform with the EU legislation on the general data protection regulation and local ethical regulations.

## Abbreviations

Aβ: Amyloid-beta
AD: Alzheimer’s disease
APOE: Apolipoprotein E
FDR: False discovery rate
GFAP: Glial fibrillary acidic protein
IS: Interdaily stability
IV: Intradaily variability
NfL: Neurofilament light chain
p-tau_181_: Tau phosphorylated at threonine 181
p-tau_231_: Tau phosphorylated at threonine 231
t-tau: Total tau
