# Supplementary material for: Sex differences in the relationships between 24-h rest-activity patterns and plasma markers of Alzheimer’s disease pathology
Source: Alzheimers Res Ther. 2024 Dec 30;16:277. doi: 10.1186/s13195-024-01653-y (PMC11684129; doi:10.1186/s13195-024-01653-y)
Supplement: Supplementary file 1 — Supplementary Material 1 [file 13195_2024_1653_MOESM1_ESM.docx]

**Additional File**

**Sex differences in the relationships between 24-h rest-activity patterns and plasma markers of Alzheimer’s disease pathology**

Maxime Van Egroo, Elise Beckers, Nicholas J. Ashton, Kaj Blennow, Henrik Zetterberg, Heidi I.L. Jacobs

**Supplementary Table 1.** Statistical outputs of the linear regression models with demographic measures as predictors and actigraphy-derived intradaily variability or interdaily stability as outcome variables in the whole study sample (N = 92).

**Supplementary Table 2.** Statistical outputs of the linear regression models with demographic measures as predictors and Alzheimer’s disease-related biomarkers as outcome variables in the whole study sample (N = 92).

**Supplementary Table 3.** Statistical outputs of the sensitivity analysis testing the relationships between plasma Alzheimer’s disease-related biomarkers and actigraphy derived 24-h rest-activity rhythm fragmentation (intradaily variability, top) or stability (interdaily stability, bottom), after accounting for *APOE* genotype.

**Supplementary Table 4.** Statistical outputs of the sensitivity analysis testing the relationships between plasma Alzheimer’s disease-related biomarkers and actigraphy derived 24-h rest-activity rhythm fragmentation (intradaily variability, top) or stability (interdaily stability, bottom), after accounting for depression rating.

**Supplementary Table 5.** Statistical outputs of the sensitivity analysis testing the relationships between plasma Alzheimer’s disease-related biomarkers and actigraphy derived 24-h rest-activity rhythm fragmentation (intradaily variability, top) or stability (interdaily stability, bottom), after accounting for self-reported symptoms of possible sleep apnea.

**Supplementary Table 6.** Statistical outputs of the sensitivity analysis testing sex differences between plasma Alzheimer’s disease-related biomarkers (predictors) and outcome measures of actigraphy derived 24-h rest-activity rhythm fragmentation (intradaily variability, top) or stability (interdaily stability, bottom), after accounting for sex interaction with *APOE* genotype.

**Supplementary Table 7.** Statistical outputs of the sensitivity analysis testing sex differences between plasma Alzheimer’s disease-related biomarkers (predictors) and outcome measures of actigraphy derived 24-h rest-activity rhythm fragmentation (intradaily variability, top) or stability (interdaily stability, bottom), after accounting for sex interaction with depression rating.

**Supplementary Table 8.** Statistical outputs of the sensitivity analysis testing sex differences between plasma Alzheimer’s disease-related biomarkers (predictors) and outcome measures of actigraphy derived 24-h rest-activity rhythm fragmentation (intradaily variability, top) or stability (interdaily stability, bottom), after accounting for sex interaction with self-reported symptoms of sleep apnea.

**Supplementary Table 9.** Statistical outputs of the sensitivity analysis testing sex differences between plasma Alzheimer’s disease-related biomarkers (predictors) and outcome measures of actigraphy derived 24-h rest-activity rhythm fragmentation (intradaily variability, top) or stability (interdaily stability, bottom), after stratifying the group of women into younger *vs.* older women using a median split based on age (59 years).

**Supplementary Figure 1.** Sex differences in the associations between actigraphy-derived 24-h rest-activity rhythm fragmentation (intradaily variability, IV) or stability (interdaily stability, IS) and plasma levels of neurofilament light chain (NfL), glial fibrillary acidic protein (GFAP), tau phosphorylated at threonine 181 (p-tau_181_), and tau phosphorylated at threonine 231 (p-tau_231_), after further stratifying the group of women into younger *vs.* older women using a median split based on age (59 years).

**Supplementary Table 1.** Statistical outputs of the linear regression models with demographic measures as predictors and actigraphy-derived intradaily variability or interdaily stability as outcome variables in the whole study sample (N = 92).

|  | Intradaily variability | | Interdaily stability | |
| --- | --- | --- | --- | --- |
|  | ***t*** | ***P*** | ***t*** | ***P*** |
| Age | 1.87 | 0.06 | 3.53 | **<0.001** |
| Male sex | 2.57 | **0.01** | -1.82 | 0.07 |
| Education | 0.78 | 0.44 | -1.91 | 0.06 |
| Body mass index | 0.01 | 0.99 | -0.89 | 0.38 |
| Actigraphic recording duration | 1.68 | 0.10 | -1.82 | 0.07 |

**Supplementary Table 2.** Statistical outputs of the linear regression models with demographic measures as predictors and Alzheimer’s disease-related biomarkers as outcome variables in the whole study sample (N = 92).

|  | NfL | | GFAP | | Aβ_42/40_ | | t-tau^a^ | | p-tau_181_^a^ | | p-tau_231_ | |
| --- | --- | --- | --- | --- | --- | --- | --- | --- | --- | --- | --- | --- |
|  | ***t*** | ***P*** | ***t*** | ***P*** | ***t*** | ***P*** | ***t*** | ***P*** | ***t*** | ***P*** | ***t*** | ***P*** |
| Age | 8.03 | **<0.001** | 5.31 | **<0.001** | -3.13 | **0.002** | -1.10 | 0.27 | 2.58 | **0.01** | -0.39 | 0.70 |
| Male sex | 0.33 | 0.74 | -0.83 | 0.41 | -0.14 | 0.89 | -1.10 | 0.28 | 2.20 | **0.03** | 1.39 | 0.17 |
| Education | 0.06 | 0.95 | 3.08 | 0.40 | 0.01 | 0.99 | -0.80 | 0.43 | 0.12 | 0.90 | -1.22 | 0.23 |
| Body mass index | -2.04 | **0.04** | -2.20 | **0.03** | 0.28 | 0.78 | -0.01 | 0.99 | -1.66 | 0.10 | -1.82 | 0.07 |

^a^ Plasma values missing for n = 6 individuals.

**Supplementary Table 3.** Statistical outputs of the sensitivity analysis testing the relationships between plasma Alzheimer’s disease-related biomarkers and actigraphy derived 24-h rest-activity rhythm fragmentation (intradaily variability, top) or stability (interdaily stability, bottom), after accounting for *APOE* genotype.

|  | Intradaily variability | | | | | | | | | | | |  |
| --- | --- | --- | --- | --- | --- | --- | --- | --- | --- | --- | --- | --- | --- |
|  | **NfL** | | **GFAP** | | **Aβ_42/40_** | | **t-tau** | | **p-tau_181_** | | **p-tau_231_** | | |
|  | ***t*** | ***P*** | ***t*** | ***P*** | ***t*** | ***P*** | ***t*** | ***P*** | ***t*** | ***P*** | ***t*** | ***P*** | |
| Plasma AD biomarker | 4.17 | **<0.001** | 2.42 | **0.02** | -1.84 | 0.07 | 0.95 | 0.35 | -0.78 | 0.44 | 0.39 | 0.70 | |
| Age | -1.07 | 0.29 | 0.56 | 0.57 | 1.26 | 0.21 | 1.40 | 0.17 | 1.48 | 0.14 | 1.55 | 0.12 | |
| Male sex | 2.62 | **0.01** | 2.74 | **0.008** | 2.51 | **0.01** | 2.14 | **0.04** | 2.13 | **0.04** | 2.20 | **0.03** | |
| Education | 0.75 | 0.45 | 0.88 | 0.38 | 0.70 | 0.49 | 0.75 | 0.46 | 0.63 | 0.53 | 0.81 | 0.42 | |
| Body mass index | 0.75 | 0.46 | 0.45 | 0.66 | -0.04 | 0.97 | 0.49 | 0.63 | 0.35 | 0.73 | 0.60 | 0.55 | |
| Actigraphic recording duration | 1.61 | 0.11 | 1.85 | 0.07 | 1.67 | 0.10 | 2.70 | **0.009** | 2.92 | **0.005** | 1.95 | 0.06 | |
| *APOE* ε4 carrier | 0.42 | 0.68 | 0.60 | 0.55 | 0.46 | 0.64 | 0.95 | 0.35 | 1.05 | 0.30 | 0.69 | 0.49 | |
|  |  |  |  |  |  |  |  |  |  |  |  |  | |
|  | **Interdaily stability** | | | | | | | | | | | |  |
|  | **NfL** | | **GFAP** | | **Aβ_42/40_** | | **t-tau** | | **p-tau_181_** | | **p-tau_231_** | | |
|  | ***t*** | ***P*** | ***t*** | ***P*** | ***t*** | ***P*** | ***t*** | ***P*** | ***t*** | ***P*** | ***t*** | ***P*** | |
| Plasma AD biomarker | -2.13 | **0.04** | -0.51 | 0.61 | -0.32 | 0.75 | -0.20 | 0.84 | -0.28 | 0.78 | -0.65 | 0.52 | |
| Age | 3.84 | **<0.001** | 5.26 | **<0.001** | 2.85 | **0.006** | 2.86 | **0.006** | 2.83 | **0.006** | 3.02 | **0.003** | |
| Male sex | -1.69 | 0.10 | -1.72 | 0.09 | -1.68 | 0.10 | -1.56 | 0.12 | -1.47 | 0.15 | -1.71 | 0.09 | |
| Education | -1.74 | 0.09 | -1.71 | 0.09 | -1.65 | 0.10 | -1.83 | 0.07 | -1.83 | 0.07 | -1.88 | 0.06 | |
| Body mass index | -1.10 | 0.28 | -0.74 | 0.46 | -0.61 | 0.54 | -0.99 | 0.32 | -1.03 | 0.31 | -0.89 | 0.38 | |
| Actigraphic recording duration | -1.75 | 0.08 | -1.85 | 0.07 | -1.83 | 0.07 | -1.89 | 0.06 | -1.86 | 0.07 | -1.68 | 0.10 | |
| *APOE* ε4 carrier | -0.98 | 0.33 | -1.13 | 0.26 | -1.21 | 0.23 | -1.10 | 0.28 | -1.01 | 0.32 | -0.94 | 0.35 | |

**Supplementary Table 4.** Statistical outputs of the sensitivity analysis testing the relationships between plasma Alzheimer’s disease-related biomarkers and actigraphy derived 24-h rest-activity rhythm fragmentation (intradaily variability, top) or stability (interdaily stability, bottom), after accounting for depression rating.

|  | Intradaily variability | | | | | | | | | | | |  |
| --- | --- | --- | --- | --- | --- | --- | --- | --- | --- | --- | --- | --- | --- |
|  | **NfL** | | **GFAP** | | **Aβ_42/40_** | | **t-tau** | | **p-tau_181_** | | **p-tau_231_** | | |
|  | ***t*** | ***P*** | ***t*** | ***P*** | ***t*** | ***P*** | ***t*** | ***P*** | ***t*** | ***P*** | ***t*** | ***P*** | |
| Plasma AD biomarker | 4.21 | **<0.001** | 2.52 | **0.01** | -1.97 | 0.052 | 0.95 | 0.35 | -0.59 | 0.56 | 0.42 | 0.68 | |
| Age | -1.21 | 0.23 | 0.38 | 0.70 | 1.15 | 0.26 | 1.23 | 0.22 | 1.23 | 0.22 | 1.42 | 0.16 | |
| Male sex | 2.67 | **0.009** | 2.85 | **0.006** | 2.59 | **0.01** | 2.29 | **0.03** | 2.25 | **0.03** | 2.30 | **0.02** | |
| Education | 0.77 | 0.44 | 1.15 | 0.25 | 0.92 | 0.36 | 0.91 | 0.37 | 0.88 | 0.38 | 1.01 | 0.31 | |
| Body mass index | 0.86 | 0.39 | 0.60 | 0.55 | 0.06 | 0.97 | 0.60 | 0.55 | 0.51 | 0.61 | 0.76 | 0.45 | |
| Actigraphic recording duration | 1.59 | 0.12 | 1.89 | 0.06 | 1.79 | 0.09 | 2.64 | **0.01** | 2.82 | **0.006** | 1.05 | 0.06 | |
| Depression rating | -0.08 | 0.93 | 0.61 | 0.54 | 0.53 | 0.60 | 0.21 | 0.84 | 0.39 | 0.70 | 0.42 | 0.68 | |
|  |  |  |  |  |  |  |  |  |  |  |  |  | |
|  | **Interdaily stability** | | | | | | | | | | | |  |
|  | **NfL** | | **GFAP** | | **Aβ_42/40_** | | **t-tau** | | **p-tau_181_** | | **p-tau_231_** | | |
|  | ***t*** | ***P*** | ***t*** | ***P*** | ***t*** | ***P*** | ***t*** | ***P*** | ***t*** | ***P*** | ***t*** | ***P*** | |
| Plasma AD biomarker | -2.17 | **0.03** | -0.65 | 0.52 | -0.08 | 0.93 | -0.16 | 0.88 | -0.28 | 0.78 | -0.68 | 0.50 | |
| Age | 4.16 | **<0.001** | 3.38 | **0.001** | 3.31 | **0.001** | 3.19 | **0.002** | 2.83 | **0.006** | 3.30 | **0.001** | |
| Male sex | -1.80 | 0.08 | -1.88 | 0.06 | -1.83 | 0.07 | -1.77 | 0.08 | -1.03 | 0.31 | -1.84 | 0.07 | |
| Education | -1.93 | 0.06 | -2.03 | **0.05** | -1.97 | **0.05** | -2.13 | **0.04** | -1.86 | 0.07 | -2.15 | **0.03** | |
| Body mass index | -1.33 | 0.19 | -1.01 | 0.32 | -0.88 | 0.38 | -1.13 | 0.25 | -1.01 | 0.32 | -1.10 | 0.27 | |
| Actigraphic recording duration | -1.74 | 0.08 | -1.88 | 0.06 | -1.84 | 0.07 | -1.83 | 0.07 | -1.86 | 0.07 | -1.69 | 0.10 | |
| Depression rating | -0.30 | 0.77 | -0.61 | 0.55 | -0.55 | 0.58 | -0.68 | 0.50 | -1.01 | 0.32 | -0.68 | 0.50 | |

**Supplementary Table 5.** Statistical outputs of the sensitivity analysis testing the relationships between plasma Alzheimer’s disease-related biomarkers and actigraphy derived 24-h rest-activity rhythm fragmentation (intradaily variability, top) or stability (interdaily stability, bottom), after accounting for self-reported symptoms of possible sleep apnea.

|  | Intradaily variability | | | | | | | | | | | |  |
| --- | --- | --- | --- | --- | --- | --- | --- | --- | --- | --- | --- | --- | --- |
|  | **NfL** | | **GFAP** | | **Aβ_42/40_** | | **t-tau** | | **p-tau_181_** | | **p-tau_231_** | | |
|  | ***t*** | ***P*** | ***t*** | ***P*** | ***t*** | ***P*** | ***t*** | ***P*** | ***t*** | ***P*** | ***t*** | ***P*** | |
| Plasma AD biomarker | 4.30 | **<0.001** | 2.39 | **0.02** | -1.82 | 0.07 | 0.69 | 0.49 | -0.51 | 0.61 | 0.39 | 0.70 | |
| Age | -1.65 | 0.10 | -0.04 | 0.97 | 0.97 | 0.34 | 0.72 | 0.48 | 0.71 | 0.48 | 0.99 | 0.33 | |
| Male sex | 2.40 | **0.02** | 2.71 | **0.008** | 2.14 | **0.04** | 2.01 | **0.05** | 1.97 | **0.05** | 2.01 | **0.05** | |
| Education | 1.25 | 0.22 | 1.22 | 0.23 | 0.88 | 0.38 | 1.17 | 0.25 | 1.12 | 0.27 | 1.19 | 0.24 | |
| Body mass index | 1.17 | 0.24 | 0.76 | 0.45 | 0.51 | 0.61 | 1.07 | 0.29 | 1.02 | 0.31 | 1.13 | 0.26 | |
| Actigraphic recording duration | 0.97 | 0.33 | 1.04 | 0.30 | 1.29 | 0.20 | 2.36 | **0.02** | 2.45 | **0.02** | 1.44 | 0.15 | |
| Self-reported symptoms of sleep apnea | -0.94 | 0.35 | -0.34 | 0.74 | -0.71 | 0.48 | -0.84 | 0.40 | -0.69 | 0.49 | -0.38 | 0.71 | |
|  |  |  |  |  |  |  |  |  |  |  |  |  | |
|  | **Interdaily stability** | | | | | | | | | | | |  |
|  | **NfL** | | **GFAP** | | **Aβ_42/40_** | | **t-tau** | | **p-tau_181_** | | **p-tau_231_** | | |
|  | ***t*** | ***P*** | ***t*** | ***P*** | ***t*** | ***P*** | ***t*** | ***P*** | ***t*** | ***P*** | ***t*** | ***P*** | |
| Plasma AD biomarker | -1.95 | **0.05** | -0.56 | 0.58 | -0.36 | 0.72 | -0.33 | 0.75 | -0.28 | 0.78 | 0.38 | 0.70 | |
| Age | 3.83 | **<0.001** | 3.09 | **0.003** | 3.12 | **0.003** | 3.51 | **<0.001** | 2.83 | **0.006** | 3.14 | **0.003** | |
| Male sex | -1.64 | 0.11 | -1.73 | 0.09 | -1.68 | 0.10 | -1.19 | 0.24 | -1.47 | 0.15 | -1.80 | 0.07 | |
| Education | -2.10 | **0.04** | -2.02 | **0.05** | -1.98 | **0.05** | -2.04 | **0.05** | -1.83 | 0.07 | -2.05 | **0.04** | |
| Body mass index | -1.58 | 0.12 | -1.30 | 0.20 | -1.20 | 0.23 | -1.80 | 0.08 | -1.03 | 0.31 | -1.31 | 0.20 | |
| Actigraphic recording duration | -1.47 | 0.15 | -1.54 | 0.13 | -1.57 | 0.12 | -2.11 | **0.04** | -1.86 | 0.07 | -1.66 | 0.10 | |
| Self-reported symptoms of sleep apnea | 0.04 | 0.97 | -0.18 | 0.86 | -0.18 | 0.86 | 0.37 | 0.72 | -1.01 | 0.32 | -0.26 | 0.80 | |

**Supplementary Table 6.** Statistical outputs of the sensitivity analysis testing sex differences between plasma Alzheimer’s disease-related biomarkers (predictors) and outcome measures of actigraphy derived 24-h rest-activity rhythm fragmentation (intradaily variability, top) or stability (interdaily stability, bottom), after accounting for sex interaction with *APOE* genotype.

|  | Intradaily variability | | | | | | | | | | | |  |
| --- | --- | --- | --- | --- | --- | --- | --- | --- | --- | --- | --- | --- | --- |
|  | **NfL** | | **GFAP** | | **Aβ_42/40_** | | **t-tau** | | **p-tau_181_** | | **p-tau_231_** | | |
|  | ***t*** | ***P*** | ***t*** | ***P*** | ***t*** | ***P*** | ***t*** | ***P*** | ***t*** | ***P*** | ***t*** | ***P*** | |
| Plasma AD biomarker*Male sex | 4.19 | **<0.001** | 3.55 | **<0.001** | -1.11 | 0.27 | 0.23 | 0.82 | 3.45 | **<0.001** | 3.16 | **0.002** | |
| Plasma AD biomarker | 1.14 | 0.26 | -0.22 | 0.83 | -0.45 | 0.66 | 0.54 | 0.59 | -2.83 | **0.006** | -1.72 | 0.09 | |
| Age | -1.51 | 0.13 | 0.45 | 0.66 | 1.26 | 0.21 | 1.45 | 0.15 | 1.99 | **0.05** | 1.73 | 0.09 | |
| Male sex | -2.87 | **0.005** | -2.09 | **0.04** | 1.39 | 0.17 | 0.19 | 0.85 | -2.70 | **0.009** | -2.25 | **0.03** | |
| Education | 0.79 | 0.43 | 0.75 | 0.45 | 0.77 | 0.44 | 0.81 | 0.42 | 0.84 | 0.40 | 0.97 | 0.33 | |
| Body mass index | 0.90 | 0.37 | 0.64 | 0.53 | 0.17 | 0.87 | 0.52 | 0.60 | 0.28 | 0.78 | 0.82 | 0.42 | |
| Actigraphic recording duration | 1.39 | 0.17 | 2.25 | **0.03** | 1.61 | 0.11 | 2.70 | **0.009** | 2.12 | **0.04** | 1.42 | 0.16 | |
| *APOE* ε4 carrier | -0.15 | 0.88 | 0.23 | 0.82 | 0.20 | 0.84 | 0.01 | 0.99 | 0.14 | 0.89 | 0.11 | 0.91 | |
| *APOE* ε4 carrier*Male Sex | 0.98 | 0.33 | 0.24 | 0.81 | 0.43 | 0.67 | 0.85 | 0.40 | 0.60 | 0.55 | -0.06 | 0.96 | |
|  |  |  |  |  |  |  |  |  |  |  |  |  | |
|  | **Interdaily stability** | | | | | | | | | | | |  |
|  | **NfL** | | **GFAP** | | **Aβ_42/40_** | | **t-tau** | | **p-tau_181_** | | **p-tau_231_** | | |
|  | ***t*** | ***P*** | ***t*** | ***P*** | ***t*** | ***P*** | ***t*** | ***P*** | ***t*** | ***P*** | ***t*** | ***P*** | |
| Plasma AD biomarker*Male sex | -2.03 | **0.05** | -1.59 | 0.12 | 0.23 | 0.82 | 1.13 | 0.26 | -3.01 | **0.004** | -1.47 | 0.15 | |
| Plasma AD biomarker | -0.60 | 0.55 | 0.65 | 0.52 | -0.19 | 0.85 | -0.78 | 0.44 | 1.70 | 0.09 | 0.55 | 0.59 | |
| Age | 4.06 | **<0.001** | 2.92 | **0.004** | 2.84 | **0.006** | 2.86 | **0.006** | 2.56 | **0.01** | 2.91 | **0.005** | |
| Male sex | 1.55 | 0.13 | 1.06 | 0.29 | -0.36 | 0.72 | -1.22 | 0.23 | 2.59 | **0.01** | 1.02 | 0.31 | |
| Education | -1.85 | 0.07 | -1.71 | 0.09 | -1.73 | 0.09 | -1.79 | 0.08 | -2.11 | **0.04** | -1.98 | **0.05** | |
| Body mass index | -1.23 | 0.22 | -0.85 | 0.40 | -0.70 | 0.49 | -1.14 | 0.26 | -1.03 | 0.31 | -1.00 | 0.32 | |
| Actigraphic recording duration | -1.55 | 0.12 | -1.93 | 0.06 | -1.75 | 0.08 | -2.11 | **0.04** | -1.15 | 0.25 | -1.38 | 0.17 | |
| *APOE* ε4 carrier | 0.26 | 0.79 | 0.03 | 0.98 | -0.20 | 0.84 | 0.08 | 0.94 | 0.07 | 0.94 | 0.03 | 0.98 | |
| *APOE* ε4 carrier*Male Sex | -1.52 | 0.13 | -1.16 | 0.25 | -0.90 | 0.37 | -1.37 | 0.17 | -0.89 | 0.38 | -0.71 | 0.48 | |

**Supplementary Table 7.** Statistical outputs of the sensitivity analysis testing sex differences between plasma Alzheimer’s disease-related biomarkers (predictors) and outcome measures of actigraphy derived 24-h rest-activity rhythm fragmentation (intradaily variability, top) or stability (interdaily stability, bottom), after accounting for sex interaction with depression rating.

|  | Intradaily variability | | | | | | | | | | | |  |
| --- | --- | --- | --- | --- | --- | --- | --- | --- | --- | --- | --- | --- | --- |
|  | **NfL** | | **GFAP** | | **Aβ_42/40_** | | **t-tau** | | **p-tau_181_** | | **p-tau_231_** | | |
|  | ***t*** | ***P*** | ***t*** | ***P*** | ***t*** | ***P*** | ***t*** | ***P*** | ***t*** | ***P*** | ***t*** | ***P*** | |
| Plasma AD biomarker*Male sex | 3.90 | **<0.001** | 3.54 | **<0.001** | -1.00 | 0.32 | 0.32 | 0.75 | 3.62 | **<0.001** | 3.18 | **0.002** | |
| Plasma AD biomarker | 1.20 | 0.23 | -0.11 | 0.91 | -0.59 | 0.56 | 0.53 | 0.60 | -2.74 | **0.008** | -1.73 | 0.09 | |
| Age | -1.71 | 0.09 | 0.29 | 0.77 | 1.13 | 0.26 | 1.25 | 0.22 | 1.79 | 0.08 | 1.78 | 0.08 | |
| Male sex | -2.78 | **0.007** | -2.20 | **0.03** | 1.28 | 0.20 | 0.23 | 0.82 | -2.66 | **0.01** | -2.34 | **0.02** | |
| Education | 1.06 | 0.29 | 1.05 | 0.30 | 1.00 | 0.32 | 0.93 | 0.35 | 0.82 | 0.41 | 0.97 | 0.34 | |
| Body mass index | 1.13 | 0.26 | 0.87 | 0.38 | 0.38 | 0.71 | 0.62 | 0.54 | 0.35 | 0.73 | 0.91 | 0.37 | |
| Actigraphic recording duration | 1.46 | 0.15 | 2.28 | **0.03** | 1.63 | 0.11 | 2.49 | **0.02** | 1.96 | **0.05** | 1.40 | 0.17 | |
| Depression rating | 0.11 | 0.91 | 0.21 | 0.84 | 0.07 | 0.94 | -0.02 | 0.98 | -0.04 | 0.97 | -0.22 | 0.83 | |
| Depression rating*Male Sex | 0.65 | 0.51 | 0.52 | 0.60 | 0.51 | 0.61 | 0.32 | 0.75 | -0.18 | 0.86 | 0.29 | 0.77 | |
|  |  |  |  |  |  |  |  |  |  |  |  |  | |
|  | **Interdaily stability** | | | | | | | | | | | |  |
|  | **NfL** | | **GFAP** | | **Aβ_42/40_** | | **t-tau** | | **p-tau_181_** | | **p-tau_231_** | | |
|  | ***t*** | ***P*** | ***t*** | ***P*** | ***t*** | ***P*** | ***t*** | ***P*** | ***t*** | ***P*** | ***t*** | ***P*** | |
| Plasma AD biomarker*Male sex | -1.78 | 0.08 | -1.45 | 0.15 | -0.02 | 0.98 | 0.91 | 0.36 | -3.25 | **0.002** | -1.60 | 0.11 | |
| Plasma AD biomarker | -0.60 | 0.55 | 0.40 | 0.69 | -0.05 | 0.96 | -0.69 | 0.49 | 1.62 | 0.11 | 0.52 | 0.61 | |
| Age | 4.35 | **<0.001** | 3.43 | **<0.001** | 3.23 | **0.002** | 3.23 | **0.002** | 2.97 | **0.004** | 3.15 | **0.002** | |
| Male sex | 0.98 | 0.33 | 0.59 | 0.056 | -0.23 | 0.82 | -1.33 | 0.19 | 2.42 | **0.02** | 0.93 | 0.36 | |
| Education | -2.03 | **0.05** | -1.94 | 0.06 | -1.94 | 0.06 | -2.02 | **0.05** | -2.10 | **0.04** | -2.09 | **0.04** | |
| Body mass index | -1.32 | 0.19 | -1.03 | 0.31 | -0.83 | 0.41 | -1.17 | 0.25 | -0.98 | 0.33 | -1.09 | 0.28 | |
| Actigraphic recording duration | -1.66 | 0.10 | -1.99 | **0.05** | -1.81 | 0.07 | -1.86 | 0.07 | -1.01 | 0.32 | -1.37 | 0.18 | |
| Depression rating | -0.49 | 0.63 | -0.50 | 0.62 | -0.40 | 0.69 | -0.45 | 0.65 | -0.46 | 0.65 | -0.29 | 0.77 | |
| Depression rating*Male Sex | -0.01 | 0.99 | 0.03 | 0.98 | -0.06 | 0.96 | -0.11 | 0.92 | 0.58 | 0.56 | 0.08 | 0.93 | |

**Supplementary Table 8.** Statistical outputs of the sensitivity analysis testing sex differences between plasma Alzheimer’s disease-related biomarkers (predictors) and outcome measures of actigraphy derived 24-h rest-activity rhythm fragmentation (intradaily variability, top) or stability (interdaily stability, bottom), after accounting for sex interaction with self-reported symptoms of sleep apnea.

|  | Intradaily variability | | | | | | | | | | | |  |
| --- | --- | --- | --- | --- | --- | --- | --- | --- | --- | --- | --- | --- | --- |
|  | **NfL** | | **GFAP** | | **Aβ_42/40_** | | **t-tau** | | **p-tau_181_** | | **p-tau_231_** | | |
|  | ***t*** | ***P*** | ***t*** | ***P*** | ***t*** | ***P*** | ***t*** | ***P*** | ***t*** | ***P*** | ***t*** | ***P*** | |
| Plasma AD biomarker*Male sex | 3.77 | **<0.001** | 3.43 | **0.001** | -0.71 | 0.48 | 0.31 | 0.76 | 3.57 | **<0.001** | 3.55 | **<0.001** | |
| Plasma AD biomarker | 1.20 | 0.24 | -0.22 | 0.82 | -0.68 | 0.50 | 0.32 | 0.75 | -2.79 | **0.007** | -2.32 | **0.02** | |
| Age | -2.09 | **0.04** | -0.23 | 0.82 | 0.91 | 0.37 | 0.74 | 0.46 | 1.51 | 0.14 | 1.70 | 0.09 | |
| Male sex | -2.50 | **0.01** | -2.05 | **0.04** | 0.97 | 0.33 | 0.20 | 0.86 | -2.70 | **0.009** | -2.82 | **0.006** | |
| Education | 1.08 | 0.28 | 0.80 | 0.43 | 0.92 | 0.36 | 1.11 | 0.27 | 1.13 | 0.26 | 1.11 | 0.27 | |
| Body mass index | 1.48 | 0.14 | 0.96 | 0.34 | 0.66 | 0.51 | 1.03 | 0.31 | 0.67 | 0.51 | 1.30 | 0.20 | |
| Actigraphic recording duration | 1.03 | 0.30 | 1.33 | 0.19 | 1.29 | 0.20 | 2.22 | **0.03** | 1.85 | 0.07 | 1.12 | 0.27 | |
| Symptoms of sleep apnea | -0.88 | 0.38 | -0.65 | 0.52 | -0.61 | 0.55 | -0.86 | 0.40 | -0.12 | 0.90 | -0.34 | 0.73 | |
| Symptoms of sleep apnea*Male Sex | 0.23 | 0.82 | 0.53 | 0.60 | 0.19 | 0.85 | 0.31 | 0.76 | -0.12 | 0.91 | 0.48 | 0.63 | |
|  |  |  |  |  |  |  |  |  |  |  |  |  | |
|  | **Interdaily stability** | | | | | | | | | | | |  |
|  | **NfL** | | **GFAP** | | **Aβ_42/40_** | | **t-tau** | | **p-tau_181_** | | **p-tau_231_** | | |
|  | ***t*** | ***P*** | ***t*** | ***P*** | ***t*** | ***P*** | ***t*** | ***P*** | ***t*** | ***P*** | ***t*** | ***P*** | |
| Plasma AD biomarker*Male sex | -1.87 | 0.07 | -1.77 | 0.08 | -0.24 | 0.81 | 0.52 | 0.60 | -3.44 | **0.001** | -2.83 | **0.006** | |
| Plasma AD biomarker | -0.35 | 0.73 | 0.70 | 0.49 | -0.01 | 0.99 | -0.11 | 0.91 | 2.28 | **0.03** | 2.30 | **0.02** | |
| Age | 4.02 | **<0.001** | 3.20 | **0.002** | 3.06 | **0.003** | 3.41 | **0.001** | 2.72 | **0.008** | 2.70 | **0.009** | |
| Male sex | 1.31 | 0.19 | 1.18 | 0.24 | 0.09 | 0.93 | -0.70 | 0.49 | 2.90 | **0.005** | 2.31 | **0.02** | |
| Education | -1.90 | 0.06 | -1.67 | 0.10 | -1.81 | 0.07 | -1.91 | 0.06 | -2.12 | **0.04** | -1.95 | 0.06 | |
| Body mass index | -1.70 | 0.09 | -1.38 | 0.17 | -1.09 | 0.28 | -1.73 | 0.09 | -1.42 | 0.16 | -1.45 | 0.15 | |
| Actigraphic recording duration | -1.36 | 0.18 | -1.56 | 0.12 | -1.41 | 0.16 | -2.04 | **0.05** | -1.36 | 0.18 | -1.31 | 0.19 | |
| Symptoms of sleep apnea | 0.56 | 0.58 | 0.53 | 0.60 | 0.37 | 0.71 | -0.70 | 0.49 | 0.04 | 0.97 | 0.14 | 0.89 | |
| Symptoms of sleep apnea*Male Sex | -0.90 | 0.37 | -1.04 | 0.30 | -0.79 | 0.43 | -0.59 | 0.56 | -0.26 | 0.80 | -0.91 | 0.37 | |

**Supplementary Table 9.** Statistical outputs of the sensitivity analysis testing sex differences between plasma Alzheimer’s disease-related biomarkers (predictors) and outcome measures of actigraphy derived 24-h rest-activity rhythm fragmentation (intradaily variability, top) or stability (interdaily stability, bottom), after stratifying the group of women into younger *vs.* older women using a median split based on age (59 years). The reference group for the main effect of sex and the interaction plasma AD biomarker*sex is the group of older women.

|  | Intradaily variability | | | | | | | | | | | |  |
| --- | --- | --- | --- | --- | --- | --- | --- | --- | --- | --- | --- | --- | --- |
|  | **NfL** | | **GFAP** | | **Aβ_42/40_** | | **t-tau** | | **p-tau_181_** | | **p-tau_231_** | | |
|  | ***t*** | ***P*** | ***t*** | ***P*** | ***t*** | ***P*** | ***t*** | ***P*** | ***t*** | ***P*** | ***t*** | ***P*** | |
| Plasma AD biomarker*Male sex | 2.75 | **0.007** | 2.46 | **0.02** | -0.89 | 0.38 | 1.32 | 0.19 | 2.86 | **0.005** | 2.98 | **0.004** | |
| Plasma AD biomarker*Younger female sex | -0.10 | 0.92 | 0.22 | 0.83 | -0.78 | 0.44 | 1.44 | 0.15 | -0.78 | 0.44 | 0.49 | 0.62 | |
| Plasma AD biomarker | 1.10 | 0.28 | 0.27 | 0.78 | -0.07 | 0.95 | -0.91 | 0.36 | -1.68 | 0.10 | -1.55 | 0.13 | |
| Age | -1.28 | 0.21 | 0.91 | 0.36 | 2.02 | **0.05** | 1.90 | 0.06 | 2.39 | **0.02** | 2.84 | **0.006** | |
| Male sex | -1.50 | 0.14 | -0.88 | 0.38 | 1.39 | 0.17 | -0.61 | 0.54 | -1.72 | 0.09 | -1.86 | 0.07 | |
| Younger female sex | 0.18 | 0.86 | 0.27 | 0.79 | 1.09 | 0.28 | -0.87 | 0.39 | 1.32 | 0.19 | 0.43 | 0.66 | |
| Education | 0.84 | 0.40 | 0.81 | 0.42 | 0.67 | 0.50 | 0.73 | 0.47 | 0.85 | 0.40 | 0.95 | 0.34 | |
| Body mass index | 1.01 | 0.32 | 0.77 | 0.42 | 0.13 | 0.89 | 0.80 | 0.43 | 0.46 | 0.65 | 0.83 | 0.41 | |
| Actigraphic recording duration | 1.46 | 0.15 | 2.22 | **0.03** | 1.87 | 0.07 | 2.67 | **0.01** | 2.15 | **0.03** | 1.43 | 0.16 | |
|  |  |  |  |  |  |  |  |  |  |  |  |  | |
|  | **Interdaily stability** | | | | | | | | | | | |  |
|  | **NfL** | | **GFAP** | | **Aβ_42/40_** | | **t-tau** | | **p-tau_181_** | | **p-tau_231_** | | |
|  | *t* | *P* | *t* | *P* | *t* | *P* | *t* | *P* | *t* | *P* | *t* | *P* | |
| Plasma AD biomarker*Male sex | -2.47 | **0.02** | -0.87 | 0.39 | -0.27 | 0.78 | -1.22 | 0.23 | -2.47 | **0.02** | -2.38 | **0.02** | |
| Plasma AD biomarker*Younger female sex | 0.89 | 0.38 | -0.04 | 0.97 | -0.04 | 0.97 | -2.89 | **0.005** | 0.89 | 0.38 | -1.63 | 0.11 | |
| Plasma AD biomarker | -0.25 | 0.80 | 0.09 | 0.93 | 0.04 | 0.97 | 1.93 | 0.06 | 0.71 | 0.48 | 1.58 | 0.12 | |
| Age | 3.56 | **<0.001** | 2.55 | **0.01** | 1.95 | 0.06 | 2.61 | **0.01** | 2.14 | **0.04** | 1.81 | 0.07 | |
| Male sex | 1.05 | 0.30 | 0.02 | 0.99 | -0.06 | 0.95 | 0.79 | 0.43 | 1.74 | 0.09 | 1.56 | 0.12 | |
| Younger female sex | 0.61 | 0.54 | -0.24 | 0.81 | -0.18 | 0.86 | 2.54 | **0.01** | -1.06 | 0.29 | 0.95 | 0.34 | |
| Education | -2.08 | **0.04** | -1.82 | 0.07 | -1.75 | 0.08 | -1.70 | 0.09 | -2.13 | **0.04** | -2.22 | **0.03** | |
| Body mass index | -1.29 | 0.20 | -1.06 | 0.29 | -0.77 | 0.44 | -1.76 | 0.08 | -1.22 | 0.23 | -1.14 | 0.26 | |
| Actigraphic recording duration | -1.40 | 0.17 | -1.93 | 0.06 | -1.81 | 0.07 | -1.97 | **0.05** | -1.07 | 0.29 | -1.19 | 0.24 | |

**Supplementary Figure 1.** Sex differences in the associations between actigraphy-derived 24-h rest-activity rhythm fragmentation (intradaily variability, IV) or stability (interdaily stability, IS) and plasma levels of neurofilament light chain (NfL), glial fibrillary acidic protein (GFAP), tau phosphorylated at threonine 181 (p-tau_181_), and tau phosphorylated at threonine 231 (p-tau_231_), after further stratifying the group of women into younger *vs.* older women using a median split based on age (59 years). Statistical models include covariates of age, sex, education, body mass index, and actigraphic recording duration. Regression lines and associated 95% confidence interval for younger female, older female, and male participants are displayed in orange, red, and blue, respectively.
